# Cytokine gene polymorphisms in Greek patients with severe asthma

**DOI:** 10.1186/2045-7022-5-S2-P9

**Published:** 2015-03-23

**Authors:** Despina Papakosta, Eirini Kontakioti, Kalliopi Domvri, Evagelia Fouka, Despina Ioannidou, Konstantinos Porpodis, Michail Daniilidis, Konstantinos Zarogoulidis

**Affiliations:** 1 Pulmonary Department Aristotle University of Thessaloniki, Thessaloniki, Greece; 2 Aristotle University of Thessaloniki, Pulmonary Department, Thessaloniki, Greece; 3 Aristotle University of Thessaloniki, 1st Department of Internal Medicine, Thessaloniki, Greece

## Background

The aim of the present study was to investigate the cytokine genetic profile in patients with severe asthma.

## Method

The study population consisted of 52 patients, 30 with allergic asthma, mean age 35,2±14,51 years (Group A), 22 patients with non-allergic asthma, mean age 47,1±16,3 years (Group B) and 21 healthy controls, mean age 36,6±10,5 years (Group C). 29 patients had mild, 10 moderate and 12 severe asthma. For the immunogenetic study DNA was extracted from the patients' peripheral blood samples. Determination of gene polymorphisms were performed by PCR using the INVITROGEN kit.

## Results

Analysis of IL-1 polymorphisms and in particular IL-1β (+3962) showed statistically significant differences between Group A-B (p=0.029) and Group B-C (p=0.024) respectively. Analysis of IL4 -1098 polymorphisms showed an increased frequency of guanine in Group A (p=0.036) when compared to Group C whereas for IL4 -590, CC genotype showed an increased frequency in Group C and C/T in Group A (p=0.003), suggesting possible association between thymine and risk of asthma development. Regarding IL-10 the diplotype GCC/ACC was more frequent in Group A when compared with Group (p=0,038). The TGF-β1 diplotype CG/CC showed increased frequencies in comparison with Group B and Group C (p=0,041 και p=0,012 αντίστοιχα). Analysis of TNFα-238 in G/G and A/G genotypes showed statistically significant differences between the Group A-C, (p=0.008) and Group B-C (p=0.001). G/G showed an increased frequency in Group C and A/G in Groups A and B. Besides, analysis of IFN-γ showed that the AA genotype was associated with Group C whereas the genotype AT was associated with risk of development of non allergic asthma (p=0,006). Regarding asthma severity, severe asthma compared with mild asthma, was associated with the genotype C/T of IL-1α and IL1β+3962 (p=0.019 και p=0.025 respectively), with the IL-4TTT/GCC diplotype, with the IL10GCC/ACC και IL10GCC/ATA diplotypes (p=0.045) and with the IL-12AC genotype ((p=0.046).

